# Point-of-Care Resuscitative Echocardiography Diagnosis of Intracardiac Thrombus during cardiac arrest (PREDICT Study): A retrospective, observational cohort study

**DOI:** 10.1016/j.resplu.2022.100218

**Published:** 2022-03-11

**Authors:** Vincent Lau, Michael Blaszak, Jason Lam, Mark German, Frank Myslik

**Affiliations:** a Department of Critical Care Medicine, Faculty of Medicine and Dentistry, University of Alberta, Edmonton, Alberta, Canada; b Division of Emergency Medicine, Department of Medicine, Schulich School of Medicine & Dentistry, Western University, London, Ontario, Canada; c Department of Anesthesiology, Pharmacology and Therapeutics, University of British Columbia, Vancouver, British Columbia, Canada

**Keywords:** Intracardiac thrombus, Point-of-care ultrasound, Echocardiography, Resuscitation, Cardiac arrest, Cardiac standstill, Mortality, POCUS

## Abstract

**Background:**

Point-of-care ultrasound (POCUS) has been previously studied in cardiac arrest, without definitive markers for futile resuscitation efforts identified. Intracardiac thrombus during cardiac arrest has not been systematically studied. Our objective was to describe the incidence of intracardiac thrombus and spontaneous echo contrast found during cardiac arrest.

**Methods:**

A two hospital, retrospective, observational cohort study of 56 cardiac arrest patients who were assessed with POCUS (between January 1st, 2017 to April 30th, 2020). Eligible studies were reviewed for echocardiographic findings (e.g. presence of intracardiac thrombus or spontaneous echo contrast), baseline patient demographics, cardiac arrest-related data, and clinical outcomes. Primary outcome was in-hospital mortality.

**Results:**

Fifty-six intra-arrest POCUS echocardiograms were identified (out of 738 out-of-hospital cardiac arrests). The median patient age was 63 years (interquartile range [IQR]: 51–72), with 25% female patients, and median Charlson Comorbidity Index score of 4 (IQR: 2–6). The incidence of intracardiac thrombus was 21 out of 56 patients (38%). Time-to-new thrombus formation during cardiac arrest was approximately 6 minutes (IQR: 2-–8). All patients with intracardiac thrombus during cardiac arrest had termination of resuscitation.

**Conclusions:**

Intracardiac thrombus is potentially common during out-of-hospital cardiac arrests and was observed more frequently in those in whom termination of resuscitation was recommended. However, this is only hypothesis-generating at this time, and further study is required to determine if the presence of intracardiac thrombus may be used as a potential marker of resuscitation futility.

## Introduction

Point-of-care ultrasound (POCUS) has demonstrated utility in a number of situations for critically ill patients, including intra-arrest.1., 2. Prior literature has demonstrated cardiac standstill is highly predictive of mortality,1., 2., 3. but not necessarily futility. A recent study of POCUS echocardiography with pulseless electrical activity (PEA) or asystole found that cardiac standstill was not associated with 100% mortality, with 0.6% of patients surviving to hospital discharge.^4^ Therefore, a reliable echocardiographic marker of resuscitation futility has not been identified.

Spontaneous echo contrast (SEC) has been described in the echocardiography literature in multiple cardiac conditions suggesting a low-flow state: atrial stunning post-cardioversion for atrial fibrillation, mitral stenosis, and post mitral valve prosthesis among others.5., 6. The presence of SEC has been associated with a higher risk of embolic events in select patients.^7^ In cardiac arrest, the predictive value of SEC has not been rigorously studied. Intuitively, SEC and evidence of clot formation potentially represent sequelae of low-flow states, which may not be conducive with life, despite cardio-pulmonary resuscitation (CPR) and other adjuncts.

There is evidence of intracardiac thrombus formation during cardiac arrest, due to ceasing of blood flow during cardiac standstill.^8^ Prior animal models suggest that ventricular fibrillation (VF) can cause intracardiac thrombus formation within 6 minutes, which occurred in up to 96% of animals after 12 minutes of untreated VF. After 2 minutes of CPR, thrombus resolved in only 72% of animals.^9^ Intracardiac or intra-vascular thrombus formation during cardiac arrest has been previously described in humans.8., 10., 11. However, the effects of embolization of these intracardiac thrombi on overall clinical outcomes (e.g. mortality, neurological outcomes, systemic embolization) following cardiac arrest in humans is unknown.

The role of POCUS echocardiography in current advanced cardiac life support (ACLS) algorithms is an area of ongoing debate.8., 12., 13., 14., 15. If intracardiac thrombus is associated with universally poor patient outcomes in cardiac arrest, incorporation of POCUS into ACLS algorithms could be made routine. It is unknown what implications this would have on current algorithms, and whether interventions may still be futile in this context.^16^ However, the International Liaison Committee on Resuscitation (ILCOR) states that no isolated sonographic finding has sufficient and/or consistent sensitivity for any clinical outcome to be used as sole criterion to terminate resuscitation.17., 18. However, there may be a role for a constellation of concomitant sonographic and clinical findings (e.g. cardiac standstill and intracardiac thrombus) which may allow such prognostication, similar to multiple neurological signs and investigations required for neuro-prognostication of persistent vegetative states post-cardiac arrest.19., 20.

To this end, the objectives of this study were to describe the incidence of both SEC and intracardiac thrombus found in cardiac arrest on POCUS echocardiography, alongside its association with clinical outcomes. We present a descriptive, exploratory, retrospective, observational cohort study of cardiac standstill, spontaneous echo contrast, intracardiac thrombus formation in out-of-hospital cardiac arrest (OHCA) patients in the emergency department.

Methods

This study was reviewed by local institutional review board (Western University Research Ethics Board and Lawson Health Research Institute) and received delegated health sciences approval (REB #: 114470).

### Setting and study design

Two Canadian centers participated in this retrospective observational cohort study at London Health Sciences Centre (LHSC) in London, Ontario, Canada. Both University and Victoria Hospital are academic (Western University) tertiary care referral centers, caring for trauma patients, all surgical subspecialties, oncological, and complex medical disorders. Both sites are equipped with portable ultrasound machines (Fujifilm Sonosite, Bothell, WA, USA; Mindray Medical, Shenzen, China) with both transthoracic echocardiography (TTE) phased array and transesophageal echocardiography (TEE) probes. Following the acquisition of a POCUS study by the physician, images are saved and uploaded to the Qpath (Telexy, Maple Ridge, BC, Canada) archiving system, along with any completed report charted by the scanning physician.

We queried all eligible consecutive emergency department (ED) cardiac arrest patients who received an intra-arrest POCUS study between January 1, 2017 to April 30, 2020 under the indication of “cardiac arrest”. This study period was selected as: (1) both TTE and TEE probes became readily available at both sites (since Jan 1, 2017); (2) was prior to hospital-wide image archival upgrade and migration of the Qpath database. Patients were excluded if: age < 18 years old, no documented arrest occurred on review of the patient’s electronic chart, POCUS scans not performed during the intra-arrest period.

### Study definitions

As previously described in other literature, cardiac standstill was defined *a priori* as “the abscence of any visible movement of the myocardium, excluding movement of blood within the cardiac chambers or isolated valve movement”.^21^

Spontaneous echo contrast detection (due to low-flow intravascular states) and grading remains somewhat nuanced, depending on technical factors (e.g. gain settings). To date, there are no universal standardized method for grading assessment.^7^ Prior scoring systems have been described.^22^ Grade 0 denotes absence of SEC, while grade 4 denotes severe SEC, with the spectrum of SEC between these grades. Given categorical nuances, we chose to dichotomize SEC as presence or absence, given that intra-arrest studies must remain focused and brief to minimize CPR interruption from a pragmatic perspective.

Intracardiac thrombus was defined as a “mass of echoes in an [intracardiac chamber] with a well circumscribed, defined border…distinct (echocardiographic texture) between the underlying myocardium and thrombus.”^23^ Prior documented echocardiographic definitions and classifications served as guidance for thrombus assessment.^24^ Intracardiac thrombus cohorts were compared to other cardiac arrest patients who did not form intracardiac thrombus during the POCUS echocardiogram.

### POCUS image acquisition

POCUS ultrasound images were obtained during ED admission, and subsequent POCUS was performed through the cardiac arrest during pulse checks until the end of resuscitation, at the discretion of the attending physician. Echocardiography imaging protocols followed during cardiac arrest relied on 3-views equivalent views between TTE and TEE: (1) parasternal or sub-costal long-axis/mid-esophageal long-axis, (2) parasternal or sub-costal short-axis/transgastric short-axis, and at minimum, (3) apical 4-chamber/mid-esophageal 4-chamber to assess all chambers for thrombus). This was informed by the International Federation for Emergency Medicine Consensus Statement: Sonography in Hypotension and Cardiac Arrest (SHoC)13., 25. and/or focused TEE for emergency physicians. 26., 27. Disruptions to CPR was minimized during image acquisition (although not formally measured). Emergency physicians or trainees credentialed in POCUS performed ultrasound imaging during this study. Resuscitation followed established ACLS protocols (e.g. CPR, epinephrine, other standard medications, where applicable). Termination of resuscitation was at the discretion of individual clinicians, given the lack of published guidance on ACLS termination.^21^

### Image adjudication

All study images were reviewed by at least 2 of 3 expert POCUS echocardiographers with National Board of Echocardiography certification (examination of special competence in critical care echocardiography)^28^ to calculate inter-rater reliability for thrombus and SEC identification. If there was disagreement between diagnoses, a third reviewer would make the tiebreaker decision. These POCUS assessors were blinded to clinical outcomes during the quality assurance oversight process.

### Data collection

The Qpath database was queried for all POCUS studies in the ED who had OHCA, with and without intracardiac thrombus. Clinical outcomes data was obtained from the hospital medical record system (Cerner). The patient’s clinical outcomes were determined once the patient had a disposition from hospital (alive or deceased).

Demographic and clinical characteristic data collected included: age, sex, Charlson Comorbidity Index (CCI) score,^29^ other cardiac comorbidities, duration of cardiac arrest and time until ROSC (if available), cardiac arrest rhythm, suspected etiology of cardiac arrest, survival to hospital/ICU admission, survival-to-ward transfer and survival-to-hospital discharge, and mortality (hospital, 30 and 60-day).

Echocardiographic findings collected included: date and duration of study, POCUS exam type (TTE/TEE), duration of POCUS study, presence of intracardiac thrombus or SEC, chamber location of intracardiac thrombus or spontaneous echo contrast, incident (new) thrombus vs. prevalent (pre-existing) thrombus, time-to-first documentation of new intracardiac thrombus, and termination of resuscitation recommendations.

### Statistical analysis

Descriptive statistics were generated for demographic, echocardiographic clinical outcome variables. Continuous data were presented as means and standard deviations (SD), or medians and interquartile ranges (IQR), where applicable. Categorical data were summarized as a total number and proportion of the cohort. Data were compared (where appropriate) using a student t-test, Wilcoxon rank-sum test, Pearson’s chi-square test, Fischer’s exact test, where applicable. A *p-*value < 0.05 was considered statistically significant with 95% confidence intervals reported, if applicable. Missing data or lost-to-follow-up would be addressed by multiple imputation if missing data was >5%, where possible. If missing data >40% for individuals, we reported those patients with full complete datasets. There were no prespecified sensitivity analyses.

Inter-rater reliability for echocardiographic findings of intracardiac thrombus was calculated for Cohen’s kappa statistic, where the following interpretations were used: less than 0 (poor), 0–0.20 (slight), 0.21–0.40 (fair), 0.41–0.60 (moderate), 0.61–0.80 (substantial), 0.81–1.00 (near perfect).30., 31.

These statistical analyses were performed using SAS software, version 9.4 (SAS Institute Inc., Cary, NC, USA) or Microsoft Excel, version 14.0.6. All reporting of this observational cohort study was made in accordance with the STROBE (strengthening the reporting of observational studies in epidemiology) guidelines and checklist.^32^

## Results

### Baseline demographics and clinical characteristics

**Fig. 1:**
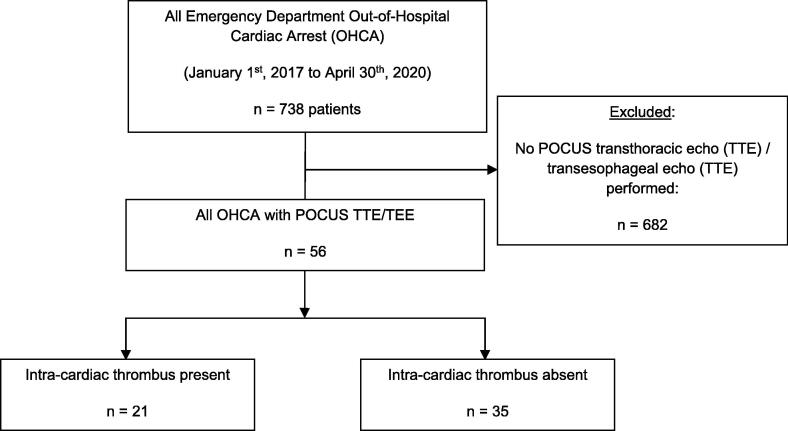
PREDICT study flow diagram. N = number, OHCA = out-of-hospital cardiac arrests, POCUS = point-of-care ultrasound, PREDICT = Point-of-care Resuscitative Echocardiography: Diagnosis of Intra-Cardiac Thrombus during Cardiac Arrest, TEE = transesophageal echocardiography, TTE = transthoracic echocardiography.

We identified 56 eligible patients who underwent OHCA with an intra-arrest POCUS ED acquired in the intra-arrest period from January 1, 2017 to April 30, 2020 (out of total 738 OHCAs, Fig. 1). There was no missing data or loss-to-follow-up with respect to outcomes.

The baseline demographics stratified by presence of intracardiac thrombus are shown in Table 1. The median age of the patients was 63 years (IQR: 51–72), 25% were female, and the median CCI index score was 4 (IQR: 2–6).

**Table 1:** Baseline Patient Demographics and Clinical Characteristics (Presence/Absence of Intra-Cardiac Thrombus).

	All Patients (n = 56)	Thrombus Present (n = 21)	Thrombus Absent (n = 35)	p-value
Age, years (median, IQR)	63 (IQR: 51, 72)	68 (IQR: 52, 82)	62 (IQR: 51, 71)	0.49
Female gender, n, (%)	14 (25%)	6 (29%)	8 (23%)	0.63
Comorbidities				
HTN, n (%)	26 (46%)	11 (52%)	15 (43%)	0.49
Dyslipidemia, n (%)	22 (39%)	10 (48%)	12 (34%)	0.32
Arrhythmia, n (%)	7 (13%)	4 (19%)	3 (9%)	0.41
Prior VTE (DVT/PE), n (%)	2 (4%)	1 (5%)	1 (3%)	1.0
Smoking, n (%)	32 (57%)	12 (57%)	20 (57%)	1.0
Prior valvulopathy, n (%)	7 (13%)	1 (5%)	6 (17%)	0.18
Charlson’s Comorbidity Index				
Age (<50y: 0, 50-59y: 1, 60-69y: 2, 70–79: 3, +80: 4), n (%)	<50 years: 11 (20%) 50–59 years: 12 (21%) 60–69 years: 13 (23%) 70–79 years: 13 (23%) 80 + years: 6 (11%)	<50 years: 4 (19%) 50–59 years: 3 (14%) 60–69 years: 5 (24%) 70–79 years: 6 (29%) 80 + years: 3 (14%)	<50 years: 7 (20%) 50–59 years: 9 (26%) 60–69 years: 8 (23%) 70–79 years: 7 (20%) 80 + years: 3 (9%)	0.82
MI, n (%)	12 (21%)	7 (33%)	5 (14%)	0.09
CHF, n (%)	4 (7%)	2 (10%)	2 (6%)	0.63
PVD, n (%)	4 (7%)	2 (10%)	2 (6%)	0.63
CVA/TIA, n (%)	5 (9%)	3 (14%)	2 (6%)	0.28
Dementia, n (%)	4 (7%)	1 (5%)	3 (9%)	0.59
COPD, n (%)	13 (23%)	9 (43%)	4 (11%)	0.01
Connective Tissue Disorder, n (%)	4 (7%)	2 (10%)	2 (6%)	0.63
Peptic ulcer disease, n (%)	7 (13%)	1 (5%)	6 (17%)	0.24
Liver disease (mild: 1, moderate-severe: 3), n (%)	None: 51 (91%) Mild: 2 (4%) Mod-severe: 3 (5%)	None: 19 (91%) Mild: 0 (0%) Mod-severe: 2 (10%)	None: 32 (91%) Mild: 2 (6%) Mod-severe: 1 (3%)	0.32
Diabetes (uncomplicated: 1, end-organ damage: 2), n (%)	None: 43 (77%) Uncomplicated: 8 (14%) End-organ Damage: 5 (9%)	None: 16 (76%) Uncomplicated: 2 (10%) End-organ Damage: 3 (14%)	None: 27 (77%) Uncomplicated: 6 (17%) End-organ Damage: 2 (6%)	0.45
Hemiplegia, n (%)	1 (2%)	0 (0%)	1 (3%)	1.0
CKD (mild: 1, mod-severe: 2), n (%)	None: 53 (95%) Mild: 2 (4%) Mod-severe: 1 (2%)	None: 21 (94%) Mild: 0 (0%) Mod-severe: 0 (0%)	None: 32 (91%) Mild: 2 (6%) Mod-severe: 1 (3%)	0.39
Solid tumour (localized: 2, metastatic: 6), n (%)	None: 50 (89%) Localized: 4 (7%) Metastatic: 2 (4%)	None: 19 (91%) Localized: 2 (10%) Metastatic: 0 (0%)	None: 31 (89%) Localized: 2 (6%) Metastatic: 2 (6%)	0.48
Leukemia, n (%)	1 (2%)	1 (5%)	0 (0%)	0.38
Lymphoma, n (%)	0 (0%)	0 (0%)	0 (0%)	1.0
AIDS, n (%)	0 (0%)	0 (0%)	0 (0%)	1.0
Charlson’s Comorbidity Index (total score), (median, IQR)	4 (IQR: 2, 6)	4 (IQR: 2, 6)	3 (IQR: 1, 6)	0.69

AIDS = acquired immunodeficiency syndrome, CHF = congestive heart failure, CKD = chronic kidney disease, COPD = chronic obstructive pulmonary disease, CVA = cerebral vascular accident, DVT = deep venous thrombosis, HTN = hypertension, IQR = interquartile range, MI = myocardial infarction, PE = pulmonary embolism, PEA = pulseless electrical activity, PVD = peripheral vascular disease, SD = standard deviation, TIA = transient ischemic attack, VF = ventricular fibrillation, VT = ventricular tachycardia, VTE = venous thromboembolism.

Characteristics of cardiac arrest are presented in Table 2. There were 27 patients (48%) presenting with PEA/asystole, 10 (18%) with VT/VF, and 19 (34%) patients with an unknown/undocumented initial rhythm during their cardiac arrest. Median time of known cardiac arrest duration was 30 minutes (IQR: 30–30) in the thrombus group, and 50 minutes (IQR: 43–60) in the no thrombus group.

**Table 2:** Cardiac arrest characteristics and echocardiographic findings.

Variable	Thrombus present	Thrombus absent	p-value
Details of cardiac arrest			
Unknown rhythm, n (%)	8/21 (38%)	11/35 (31%)	0.61
PEA/Asystole, n (%)	8/21 (38%)	19/35 (54%)	0.28
VT/VF, n (%)	5/21 (24%)	5/35 (14%)	0.37
Suspected etiology of cardiac arrest			
Unknown/undocumented NYD, n (%)	16/21 (76%)	16/35 (46%)	0.03
Overdose, n (%)	1/21 (5%)	2/35 (6%)	0.88
Ischemia/ myocardial infarction, n (%)	2/21 (10%)	3/35 (9%)	0.90
Aortic aneurysm/dissection, n (%)	1/21 (5%)	1/35 (3%)	0.71
Hemorrhage shock, n (%)	0/21 (0%)	4/35 (11%)	0.29
Pulmonary embolism, n (%)	0/21 (0%)	2/35 (6%)	0.52
Hyperkalemia, n (%)	0/21 (0%)	1/35 (3%)	1.0
Septic shock, n (%)	0/21 (0%)	1/35 (3%)	1.0
Other, n (%)	0/21 (0%)	1/35 (3%)	1.0
Hypoxemic respiratory failure, n (%)	1/21 (5%)	4/35 (11%)	0.64

Unknown total downtime, n (%)	20/21 (95%)	22/35 (63%)	0.007
Known total downtime, n (%)	1/21 (5%)	13/35 (37%)	
Duration of resuscitation, minutes, (median, IQR)	30 (IQR: 30, 30) N = 1	50 (IQR: 43, 60) N = 13	N/A
POCUS characteristics/findings:			
Exam type (TTE/TEE)	TTE: 5/21 (24%) TEE: 16/21 (76%)	TTE: 8/35 (23%) TEE: 27/35 (77%)	0.93
Duration of POCUS (minutes, median: IQR)	6 (IQR: 2, 13) N = 17	7 (IQR: 3, 12) N = 19	0.69
Cardiac standstill present, n (%)	19/21 (91%)	16/35 (46%)	0.0008
Spontaneous echo contrasts present during arrest, n (%)	19/21 (91%)	14/35 (40%)	0.0002
Intra-cardiac thrombus present at POCUS initiation (first image), n (%)	12/21 (57%)	N/A	N/A
Intra-cardiac thrombus formation during POCUS (subsequent images), n (%)	9/21 (43%)	N/A	
Time to documentation of new intra-cardiac thrombus during cardiac arrest (minutes, median: IQR)	6 (IQR: 2,8) N = 8	N/A	N/A
Location of intra-cardiac thrombus:			
LA, n (%)	15/21 (71%)	N/A	N/A
LV, n (%)	17/21 (81%)	N/A	N/A
RA, n (%)	18/21 (86%)	N/A	N/A
RV, n (%)	17/21 (81%)	N/A	N/A

CI = confidence interval, ED = emergency department, ICU = intensive care unit, IQR = interquartile range, LA = left atrium, LV = left ventricle, N/A = not applicable/not available, NYD = not yet diagnosed, PEA = pulseless electrical activity, POCUS = point-of-care ultrasonography; RA = right atrium, ROSC = return of spontaneous circulation, RV = right ventricle, SD = standard deviation, TEE = transesophageal echocardiography, TOR = termination of resuscitation, TTE = transthoracic echocardiography, VF = ventricular fibrillation, VT = ventricular tachycardia.

There were significant differences between the following cardiac arrest characteristics for thrombus presence vs. absence: unknown/undocumented etiology of cardiac arrest (16/21 patients, 76% vs. 16/35 patients, 46%, p = 0.03) and unknown total downtime (20/21 patients, 95% vs. 22/35 patients, 63%, p = 0.007).

### Echocardiographic findings and inter-rater reliability

Echocardiographic findings are presented in Table 2. Type of POCUS study performed was majority 76% TEE, with 24% TTE. Median duration of POCUS study was 6 minutes (IQR: 2–13) in the thrombus group versus 7 minutes (IQR: 3–12) in the no thrombus group.

**Fig. 2:**
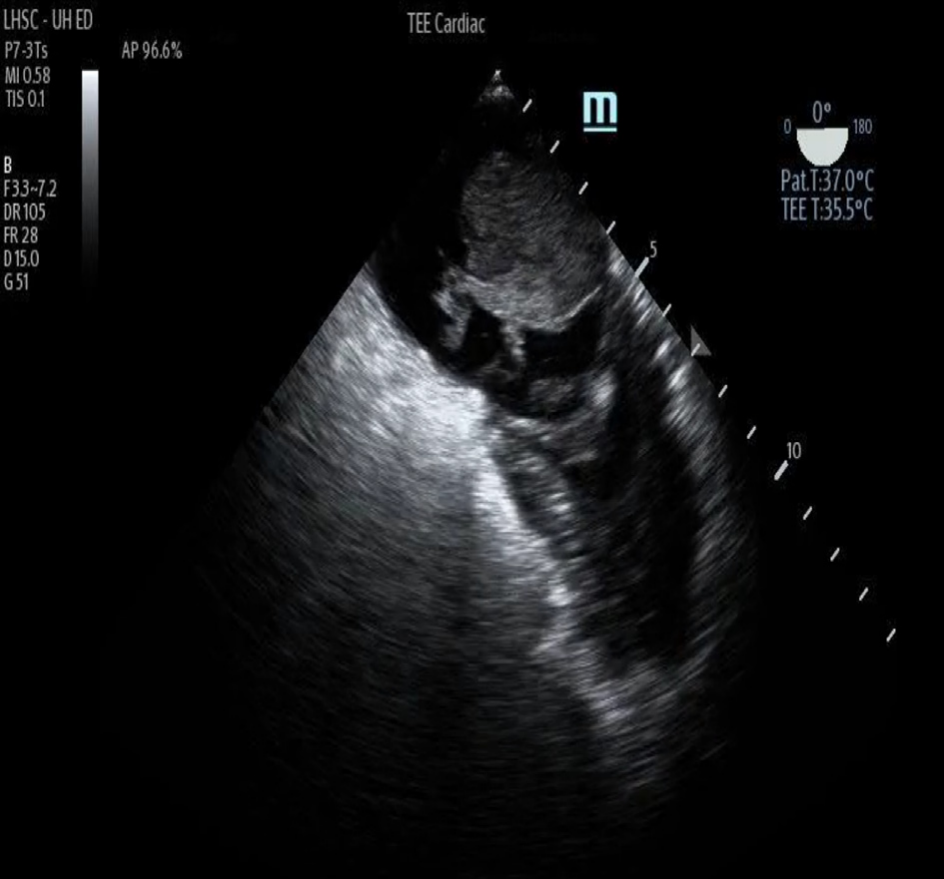
Intracardiac thrombus during cardiac arrest (still image).

Intracardiac thrombus was present in 21 of 56 patients (38%) (Fig. 2 & Supplemental Video 1), while SEC was found in 33 of 56 patients (59%), while cardiac standstill was found in 35/56 patients (63%). Pre-existing intracardiac thrombus was observed in 12/21 patients (57%), while new thrombus formation during cardiac arrest was observed in 9/21 patients (43%). The median time to new documentation of intracardiac thrombus was 6 minutes (IQR: 2–8). Cardiac chamber locations for intracardiac thrombus are noted in Table 2.

We calculated a Cohen’s kappa statistic (Supplemental Table 1) between two expert echocardiographers for the outcome of presence/absence of intracardiac thrombus or SEC. The kappa statistic (k) for intracardiac thrombus was calculated to be 0.92 (95% CI: 0.82–1.00). The kappa for SEC was calculated to be 1.00 (95% CI: 1.00–1.00). This was near perfect (k ∼ 0.81–1.00) for both intracardiac thrombus and SEC.

### Clinical outcomes

Clinical outcomes associated with OHCA are presented in Table 3. Termination of resuscitation (TOR) was recommended in 33 of 56 patients (59%), 21/21 patients (100%) in the thrombus group, and 12/35 patients (34%) in the no thrombus group. In contrast, survival to ICU admission with ROSC was 0/21 patients (0%) in the thrombus group, whereas survival to ICU admission was 23/35 (66%, p < 0.00001) in the no thrombus group.

**Table 3:** Outcomes associated with cardiac arrest and associated risk factors and variables.

Variable	All cardiac arrests (N = 56)	PEA/Asystole (N = 27)	VT/VF (N = 10)	Cardiac standstill* (N = 35)	Spontaneous echo contrast* (N = 33)	Intra-cardiac thrombus* (N = 21)	No intra-cardiac thrombus* (N = 35)	p-value (thrombus vs. none)
Termination of Resuscitation, n (%)	33/56 (59%)	17/27 (63%)	6/10 (60%)	34/35 (97%)	32/33 (97%)	21/21 (100%)	12/35 (34%)	<0.00001
Survival to ROSC/ICU admission, n (%)	23/56 (41%)	7/27 (37%)	4/10% (40%)	1/35 (3%)	1/33 (3%)	0/21 (0%)	23/35 (66%)	<0.00001
Survival to ward transfer, n (%)	1/56 (2%)	0/27 (0%)	1/10 (10%)	1/35 (3%)	0/33 (0%)	0/21 (0%)	1/35 (3%)	1.0
Alive at 30 days, n (%)	1/56 (2%)	0/27 (0%)	1/10 (10%)	1/35 (3%)	0/33 (0%)	0/21 (0%)	1/35 (3%)	1.0
Alive at 60 days, n (%)	1/56 (2%)	0/27 (0%)	1/10 (10%)	1/35 (3%)	0/33 (0%)	0/21 (0%)	1/35 (3%)	1.0
Survival to hospital discharge, n (%)	1/56 (2%)	0/27 (0%)	1/10 (10%)	1/35 (3%)	0/33 (0%)	0/21 (0%)	1/35 (3%)	1.0
Survival to discharge with good neurological outcome (GCS 15), n (%)	1/56 (2%)	0/27 (0%)	1/10 (10%)	0/35 (0%)	0/33 (0%)	0/21 (0%)	1/35 (3%)	1.0
In-hospital mortality, n (%)	55/56 (98%)	0/27 (0%)	9/10 (90%)	34/35 (97%)	33/33 (100%)	21/21 (100%)	34/35 (97%)	1.0

ICU = intensive care unit, GCS = Glasgow Coma Scale, PEA = pulseless electrical activity, ROSC = return of spontaneous circulation, VF = ventricular fibrillation, VT = ventricular tachycardia.

*Some patients may have multiple findings on the same echocardiogram: cardiac standstill, spontaneous echo contrast, and intra-cardiac thrombus.

## Discussion

We report a descriptive, retrospective cohort of presence versus absence of intra-cardiac thrombus on POCUS performed in the intra-arrest period. Intracardiac thrombus is common and was observed more frequently in those in whom termination of resuscitation was recommended. If intracardiac thrombus, alongside other echocardiographic findings (e.g. cardiac standstill), are to be used as a constellation of markers of resuscitation futility, further study is required.

Intracardiac or intra-vascular thrombus formation has been previously documented in the literature, but has been mostly from case reports or case-series. One case-series showed that 50% of patients developed a “gel-like” echo contrast within cardiac chambers, which was observed 20–30 minutes following initiation of CPR, with unrelenting cardiac arrest and uniformly associated with an adverse outcome.^33^ Our study is the first observational cohort study to systematically review the incidence of SEC and intracardiac thrombus in the intra-arrest period, with its possible association with poor patient outcomes. These findings potentially add robustness to the significance of thrombus detection. Both SEC and thrombus are entities along the spectrum of the coagulation process, as literature suggests SEC as a precursor to clot formation outside of cardiac arrest.^7^ Even with discrepancies in inter-rater reliability for intra-cardiac thrombus (kappa: 0.92), there was 100% agreement (kappa: 1.00) for SEC with universally poor outcomes for hospital mortality in this small study. This raises the question whether thrombus vs. SEC differentiation is of clinical importance as both demonstrate futility alongside other findings (e.g. cardiac standstill). Rather both can simply mark low-flow states in cardiac arrest that portends poor prognosis.

Blood flow stasis in cardiac arrest is the most likely mechanism for intracardiac thrombus formation, with prior time to clot formation estimated to be ∼ 6–12 minutes, with non-resolution in 28% in animal studies,^9^ which mirrors similar findings in our study. Whether clinicians felt the presence of intracardiac thrombus was indicative of poor outcome (leading to termination of resuscitation and a self-fulfilling prophecy) remains unclear. However, despite the presence of intracardiac thrombus formation during cardiac arrest, it may not necessarily be a treatable entity. Instead, it may be a marker of cardiac arrest severity and potential marker of futility. Prior randomized control trial evidence demonstrating that empiric thrombolytic therapy did not change overall mortality in out-of-hospital cardiac arrests, and was associated with higher rates of intracranial hemorrhage.^16^

If a marker of definitive resuscitation futility like intracardiac thrombus can be confirmed in larger and more prospectively designed and rigorous studies, then clinicians may finally have evidence to support earlier termination of resuscitation. As ultrasound machines become ubiquitous in the acute care setting, these findings may support POCUS incorporation into ACLS algorithms during cardiac arrest to allow the detection intracardiac thrombus and hence denote resuscitation futility. However, caution is still needed and further studies are required, as no one ultrasound finding is currently indicative of futility.17., 18. Therefore, a constellation of findings may be required to confidently terminate resuscitation based on clinical and echocardiographic findings.

There are important strengths of this study. This was the first study to systematically look at the formation of intracardiac thrombus in cardiac arrest patients, with the potential for using this finding to allow rapid prognostication during the intra- and/or immediate post-arrest period.

This study has also several important limitations. The overall sample size for intracardiac thrombus formation was small. There is inherent selection bias, as we only examined OHCA patients who received a POCUS echocardiogram under the indication of “cardiac arrest” in the ED. This may have resulted in missing otherwise eligible patients if providers did not record their studies and/or document the indication. There is also potential for confirmation bias, as physicians were not blinded to the real-time findings of the ultrasound, with the possibility of termination of resuscitation becoming a self-fulfilling prophecy. There was also missing data, with 60% of patient’s total duration of cardiac arrest being unknown, and 38% missing initial underlying rhythm (inherent problem in cardiac arrest research if patients are “found down”). Given the retrospective cohort analysis, there was also a lack of time-dependent variables. For future prospective, observational cohort studies of intracardiac thrombus in cardiac arrest, data collection should include Utstein cardiac arrest variables,^34^ and that adjustment for those variables should be performed. The two-center nature of our study limits its generalizability. The highly structured and closely supervised critical care ultrasound program at Western University has a rigorous quality assurance pathway for all cardiac arrest POCUS echocardiograms, which is not standard across other hospital systems.

Although these findings of this study are novel, they should not be considered definitive for markers of futility. Future work should focus on confirming these findings in a larger, robust sample of cardiac arrest patients. Assessment of neurological function post-arrest and health-related quality-of-life would be important to measure, alongside mortality outcomes for survivors to discharge. The quality of evidence would need to improve before definitive recommendations for routine POCUS incorporation into the ACLS algorithms. These findings should not be considered definitive, only hypothesis-generating, and interpreted with caution and in context. Furthermore, POCUS should be investigated to attempt improvements of cardiac arrest outcomes, by ruling in or out treatable conditions.

## Conclusion

Intracardiac thrombus is potentially common during out-of-hospital cardiac arrests and was observed more frequently in those in whom termination of resuscitation was recommended, whereby these findings are only hypothesis-generating at this time. A constellation of findings (e.g. cardiac standstill, SEC or intracardiac thrombus) may be required to develop definitive markers of resuscitation futility. Further studies with larger sample sizes and the assessment of both morbidity (e.g. poor neurological outcomes) and mortality need to be conducted prior to strong inferences or definitive recommendations being made.

## Author contributions

Vincent Lau, Michael Blaszak, Jason Lam, Mark German, Frank Myslik have: (1) made substantial contributions to conception and design, acquisition of data, analysis and interpretation of data; (2) drafted the submitted article and revised it critically for important intellectual content, and (3) provided final approval of the version to be published.

Conception: Lau, Blaszak, German, Myslik

Background: Lau, Blaszak, Lam, German, Myslik

Design: Lau, Blaszak, German, Myslik

Acquisition of data: Lau, Blaszak, Lam. German, Myslik

Analysis of data: Lau, Blaszak, Lam. German, Myslik

Drafting the manuscript: Lau, Blaszak, Lam. German, Myslik

Revising the manuscript: Lau, Blaszak, Lam. German, Myslik

## Institutional Research Ethics Board

Lawson # 114470.

## Declaration of Competing Interest

The authors declare that they have no known competing financial interests or personal relationships that could have appeared to influence the work reported in this paper.
