# Supplementary material for: Point-of-Care Resuscitative Echocardiography Diagnosis of Intracardiac Thrombus during cardiac arrest (PREDICT Study): A retrospective, observational cohort study
Source: Resusc Plus. 2022 Mar 11;10:100218. doi: 10.1016/j.resplu.2022.100218 (PMC8921470; doi:10.1016/j.resplu.2022.100218)
Supplement: Supplementary data 1 [file mmc1.docx]

**Supplemental Table 1:** Inter-rater reliability (kappa) of intra-cardiac thrombus and spontaneous echo contrast (SEC)

| Inter-relater reliability | Rater #2 | | |
| --- | --- | --- | --- |
| Rater #1 | Thrombus present | Thrombus absent | Total |
| Thrombus present | 20 | 1 | 21 |
| Thrombus absent | 1 | 34 | 35 |
| Total | 21 | 35 | 56 |
|  |  |  |  |
| Rater agreement (observed) | 20 | 34 | 54 |
| Rater agreement by chance (expected) | 7.88 | 21.88 | 29.75 |
|  |  |  |  |
|  | Kappa | 95% CI (lower limit) | 95% CI (upper limit) |
| Cohen’s kappa statistic | 0.92 | 0.82 | 1.00 |
|  |  |  |  |
| Inter-relater reliability (SEC) | Rater #2 | | |
| Rater #1 | SEC present | SEC absent | Total |
| SEC present | 33 | 0 | 33 |
| SEC absent | 0 | 23 | 23 |
| Total | 33 | 23 | 56 |
|  |  |  |  |
| Rater agreement (observed) | 33 | 23 | 56 |
| Rater agreement by chance (expected) | 19.45 | 9.45 | 28.89 |
|  |  |  |  |
|  | Kappa | 95% CI (lower limit) | 95% CI (upper limit) |
| Cohen’s kappa statistic | 1.00 | 1.00 | 1.00 |

CI = confidence interval, SEC = spontaneous echo contrast
